# Effectiveness of edoxaban in portal vein thrombosis associated with liver cirrhosis

**DOI:** 10.1038/s41598-024-60235-y

**Published:** 2024-05-11

**Authors:** Tomoko Tadokoro, Joji Tani, Takushi Manabe, Kei Takuma, Mai Nakahara, Kyoko Oura, Shima Mimura, Koji Fujita, Takako Nomura, Asahiro Morishita, Hideki Kobara, Takashi Himoto, Masafumi Ono, Tsutomu Masaki

**Affiliations:** 1 Department of Gastroenterology and Neurology, Kagawa University School of Medicine, 1750-1 Ikenobe, Miki-cho, Kita-gun, Kagawa, 761-0793 Japan; 2 Gastroenterology and Hepatology, HITO Medical Center, 788‐1 Kamibun‐cho, Shikokutyuou, Ehime 799‐0121 Japan; 3 Department of Medical Technology, Kagawa Prefectural University of Health Sciences, 281-1, Hara, Mure-Cho, Takamatsu, Kagawa 761-0123 Japan; 4 Division of Innovative Medicine for Hepatobiliary and Pancreatology, Faculty of Medicine, Kagawa University School of Medicine, 1750-1 Ikenobe, Miki-cho, Kita-gun, Kagawa, 761-0793 Japan

**Keywords:** Cirrhosis, Direct-acting oral anticoagulants, Portal vein thrombosis, Warfarin, Medical research, Hepatology

## Abstract

Portal vein thrombosis (PVT) worsens the long-term prognosis of patients with cirrhosis; however, the optimal treatment remains to be determined. Reports on the efficacy of direct oral anticoagulants are increasing, and further evidence is needed. Therefore, we investigated the effectiveness of treatment with edoxaban in patients with PVT. We retrospectively reviewed the outcomes of edoxaban and warfarin as antithrombotic therapies for PVT. The median overall survival time was 4.2 years in patients with PVT, with a 1-year survival rate of 70.7% and a 5-year survival rate of 47.9%. The leading cause of death was hepatocellular carcinoma. The overall response rate for thrombolysis in the edoxaban group was 76.7% compared to 29.4% in the warfarin group, and edoxaban significantly improved PVT compared to warfarin. In addition, edoxaban provided long-term improvement of PVT. Warfarin, on the other hand, was temporarily effective but did not provide long-term benefits. The Child–Pugh and albumin-bilirubin scores did not change after edoxaban or warfarin use. No deaths occurred due to adverse events associated with edoxaban or warfarin. Edoxaban as a single agent can achieve long-term recanalization without compromising the hepatic reserves. Edoxaban is easy to initiate, even in an outpatient setting, and could become a major therapeutic agent for the treatment of PVT.

## Introduction

Portal vein thrombosis (PVT) occurs in 5–26% of patients with cirrhosis^1,2^ and is increasingly identified owing to advances in imaging studies. Although it is generally asymptomatic, except in the acute phase, PVT increases portal venous pressure and worsens liver function, esophagogastric varices, and ascites^3,4^. It is closely associated with decreased liver reserves and a poor survival prognosis^5^. The etiology of PVT is like that of venous thromboembolism, including damage to vessel walls, decreased blood flow, and hypercoagulability. In cirrhosis, anticoagulant factors such as antithrombin III (AT III), protein C, and protein S are decreased, as are liver-derived coagulation factors (such as factors II, VII, IX, and X), resulting in an imbalance that predisposes patients to thrombus formation^6^. Additionally, increased portal pressure and decreased portal blood flow due to hepatic fibrosis contribute to PVT formation. In contrast, in patients with portal vein thrombosis, anticoagulation increases the risk of bleeding and other problems because of the presence of easy hemorrhage, gastroesophageal varices, and portal hypertensive gastropathy^7^. Therefore, there is no consensus regarding the optimal treatment for PVT, even in the rapidly advancing field of cirrhosis treatment^8^. AT III is the only drug for PVT covered by insurance in Japan^9^. AT III is effective for PVT but has drawbacks, such as being limited to cases with reduced AT III, requiring intravenous infusion, and not being suitable for maintenance therapy. In addition, danaparoid sodium-based anticoagulation therapy is effective and frequently used in the treatment of PVT^10^, but as of 2023, shipments have stopped, and it is no longer available.

The therapeutic effects of direct oral anticoagulants (DOACs) on PVT have been widely reported^11^. DOACs are effective if the therapeutic dose is appropriate because they reduce the burden of conventional injections and do not require monitoring, as does warfarin. Another advantage of DOACs is that they do not have significant drug-drug interactions, even in patients with hereditary thrombophilia^12^. Edoxaban, a DOAC, is a direct-type Xa inhibitor^13,14^, approximately 50% of which is excreted through the kidneys^15^. It is not susceptible to hepatic metabolism^16^, making it safe to use in patients with cirrhosis. Past studies have demonstrated the effectiveness of edoxaban in conjunction with other agents; however, its effectiveness as a single agent requires further investigation. In this study, we investigated the effectiveness of edoxaban monotherapy for the treatment of PVT and its impact on prognosis.

## Results

### Clinical characteristics

**Figure 1:**
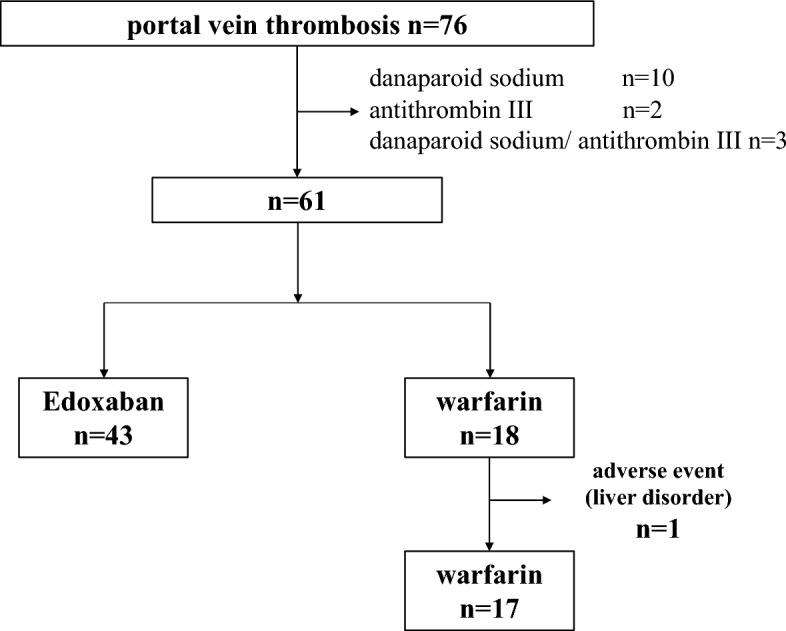
Treatment flowchart for patients with portal vein thrombosis. The choice of treatment for portal vein thrombosis was determined by the physician in charge after a comprehensive evaluation of the patient's background and other factors. Of the 76 patients with portal vein thrombosis in the setting of cirrhosis, 61 were included after excluding those treated with danaparoid sodium or antithrombin III. Edoxaban and warfarin were administered to 43 and 18 patients, respectively. One patient who used warfarin was excluded because he discontinued treatment owing to drug-induced liver injury.

**Figure 2:**
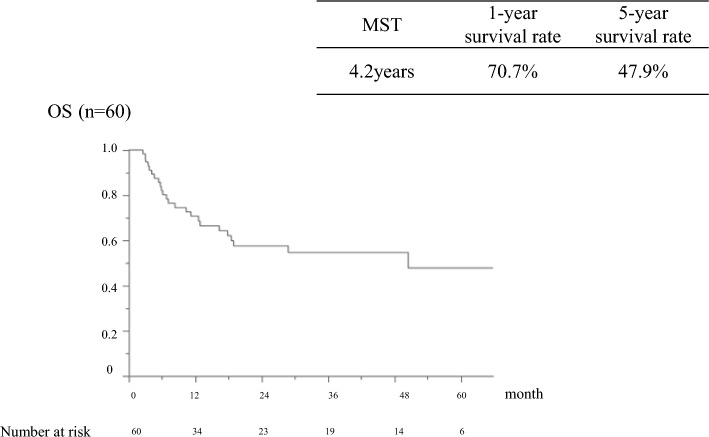
Overall survival rate of patients with portal vein thrombosis (n = 60). In the overall survival study, the median survival time (MST) was 4.2 years in patients with portal vein thrombosis, with a 1-year survival rate of 70.7% and a 5-year survival rate of 47.9%. Deaths other than liver disease were censored. There were no liver transplant patients. OS, overall survival; MST, median survival time.

Of the 61 patients treated with warfarin or edoxaban alone, 60 were included in the analysis, excluding one patient who discontinued treatment owing to hepatic dysfunction while receiving warfarin (Fig. 1). The age of the patients with PVT was 68 years (61–73) (male/female, 39/21). Cirrhosis was caused by viral hepatitis in 35 patients (12 hepatitis B and 23 hepatitis C) and non-B/C hepatitis in 25 patients. Eighteen patients had a Child–Pugh A classification, 36 had Child–Pugh B, and 6 patients had Child–Pugh C. The median ALBI score was − 1.80 (− 2.12 to − 1.46), modified ALBI grade was 1 for 6 patients, 2a for 8 patients, 2b for 35 patients, and 3 for 11 patients. Hepatocellular carcinoma (HCC) was observed in 44 patients (73%). The most common thrombus site was the main trunk of the portal vein in 44 patients (73%). Deaths were confirmed in 32 cases: 21 cases of HCC, 5 cases of hepatic failure, and 6 cases from other causes. No deaths were directly related to the anticoagulation therapy for portal vein thrombosis, such as bleeding. In the overall survival study, the median survival time (MST) was 4.2 years in patients with portal vein thrombosis, with a 1-year survival rate of 70.7% and a 5-year survival rate of 47.9% (Fig. 2).

### Effects of edoxaban or warfarin treatment on PVT

**Figure 3:**
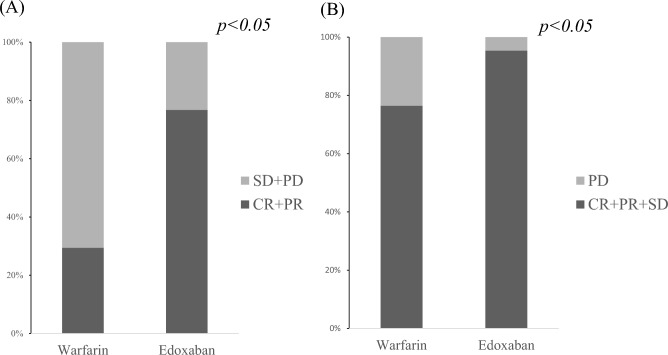
Therapeutic effectiveness of edoxaban and warfarin for portal vein thrombosis. Edoxaban significantly improves portal vein thrombosis in both ORR and DCR compared to warfarin. overall response rate (ORR: CR + PR). disease control rate (DCR: CR + PR + SD). CR, complete response; PR, partial response; SD, Stable Disease; PD, progressive disease.

The drugs used for PVT therapy were edoxaban and warfarin in 43 and 17 patients, respectively. The baseline (pretreatment) clinical characteristics are shown in Table 1. There were no significant differences in background characteristics between the groups treated with edoxaban and warfarin. The median number of days on edoxaban was 562 (212–1472), and those on warfarin was 677 (566–2145). The median PT-INR in the warfarin group was 1.95 (1.69–2.29), and 35% of patients achieved the management goal of 2.0–3.0. Edoxaban was more effective than warfarin for PVT in terms of the overall response rate (ORR; CR + PR) (*p* < 0.05) and disease control rate (DCR; CR + PR + SD) (*p* < 0.05) (Fig. 3). The ORR and DCR for the edoxaban group were 76.7% and 95.3%, respectively, while the ORR and DCR for the warfarin group were 29.4% and 76.4%, respectively. In a univariate analysis of factors associated with PVT resolution, only edoxaban use was associated, not age, gender, etiology, history of hepatocellular carcinoma, PVT localization, or time to treatment initiation (Table 2).

**Table 1:** Background, clinical features, laboratory data, and curative effect of patients with PVT treated with anticoagulants.

	Edoxaban (n = 43)	Warfarin (n = 17)	p-value
Age, years (IQR)	69 (53–74)	68 (62–73)	0.909
Gender, male/female	28/15	11/6	0.976
Etiology, HBV or HCV/NBNC	23/20	12/5	0.226
Child–Pugh grade, A/B or C (B/C)	13/25/5	5/11/1	0.950
Time from PVT to treatment, days (IQR)	21 (15–492)	13 (3–35)	0.104
Median percentage of portal vein lumen occupied, % (IQR)	100 (65.1–100)	100 (85.7–100)	0.506
Localization of PVT, MPV/others	33/10	11/6	0.342
Total bilirubin, mg/dL	0.9 (0.7–1.7)	1.1 (0.7–1.8)	0.889
Aspartate aminotransferase, IU/L	49 (36–65)	49.5 (36.3–67.3)	0.605
Alanine aminotransferase, IU/L	28 (19.5–42)	28 (21–40)	0.831
Albumin, g/dL	3.1 (2.7–3.5)	3 (2.7–3.4)	0.576
Platelet count, × 104/μL	11.3 (6.6–15.6)	9.1 (5.9–13.8)	0.398
Prothrombin activity, %	65 (53.5–84)	65 (59.0–73.0)	0.755
Activated partial thromboplastin time, ng/mL	31.5 (28.8–35)	32.9 (31.2–33.8)	0.536
Ascites, yes/no	29/14	10/7	0.528
Encephalopathy, yes/no	41/2	1/16	0.844
Esophageal varix, yes/no	35/8	15/2	0.522
Hepatocellular carcinoma, yes/no	34/9	10/8	0.247

Data are reported as number or median (IQR; interquartile range).

PVT, Portal vein thrombosis; AT III, antithrombin III; HBV, hepatitis B virus; HCC, hepatocellular carcinoma; HCV, hepatitis C; NBNC, non-B, non-C hepatitis; MPV, main portal vein; ALBI, Albumin-Bilirubin.

**Table 2:** Results of univariate analysis of factors contributing to PVT resolution.

Variables	Category	Univariate analysis		
		Odds ratio	95% confidence interval	p-value
Age	≥ 65	0.47	0.11–1.91	0.27
Gender	Male	1.55	0.45–5.28	0.49
Etiology	Viral hepatitis	2.10	0.57–7.68	0.25
History of hepatocellular carcinoma	Yes	1.13	0.30–4.30	0.85
Localization of PVT	MPV	1.14	0.34–3.73	0.83
Time to start treatment	Within 1 month	0.92	0.32–3.58	0.92
Drugs	Edoxaban	5.73	1.10–29.78	0.04

PVT, Portal vein thrombosis; AT III, antithrombin III; MPV, main portal vein.

**Figure 4:**
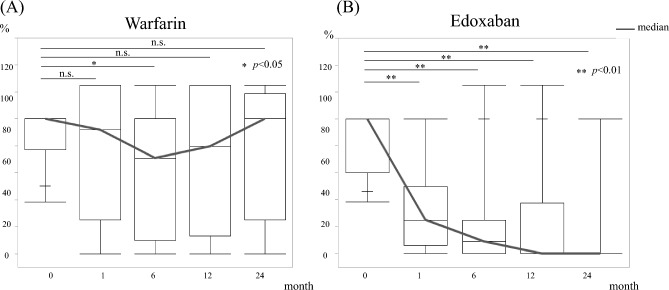
Volume change of portal vein thrombosis. The graph shows the change in portal vein thrombus occupancy in the portal vein cross-sectional area. (**A**) Warfarin shows thrombus reduction 6 months after initiation of treatment but does not show long-term reduction thereafter. (**B**) Edoxaban has a reduction in portal vein thrombosis over time.

**Figure 5:**
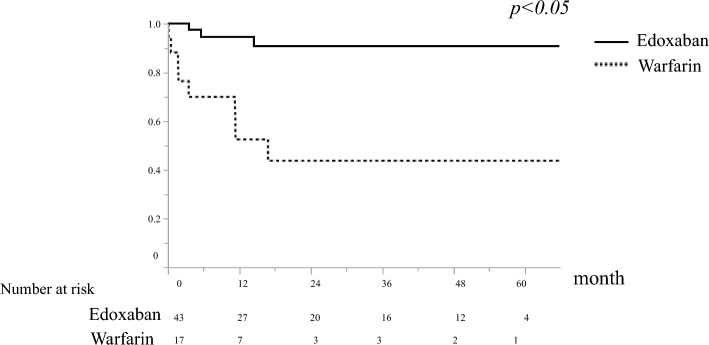
Thrombosis-free progression period. Edoxaban has significantly longer portal vein thrombus progression-free survival compared to warfarin.

Furthermore, the duration of the therapeutic effects of the two drugs was examined. Patients treated with warfarin showed thrombus reduction 6 months after initiation of treatment but did not show long-term reduction thereafter. In contrast, patients treated with edoxaban had a reduction in PVT over time (Fig. 4). In addition, edoxaban resulted in significantly longer portal vein thrombus progression-free survival than warfarin (Fig. 5). Univariate analysis of factors associated with thrombus disappearance (CR) revealed significant differences only in the use of edoxaban (Table 3).

**Table 3:** Complications observed while using warfarin or edoxaban.

complications	Warfarin n = 2 (11.1%)	Edoxaban n = 7 (16.2%)
Gastric telangiectasia		2
Ruptured esophageal varices		1
Small intestinal bleeding		1
Biliary bleeding		1
Subcutaneous hemorrhage		1
Nasal hemorrhage	1	1
Liver disorder	1	

### Safety and adverse effects of edoxaban and warfarin

**Figure 6:**
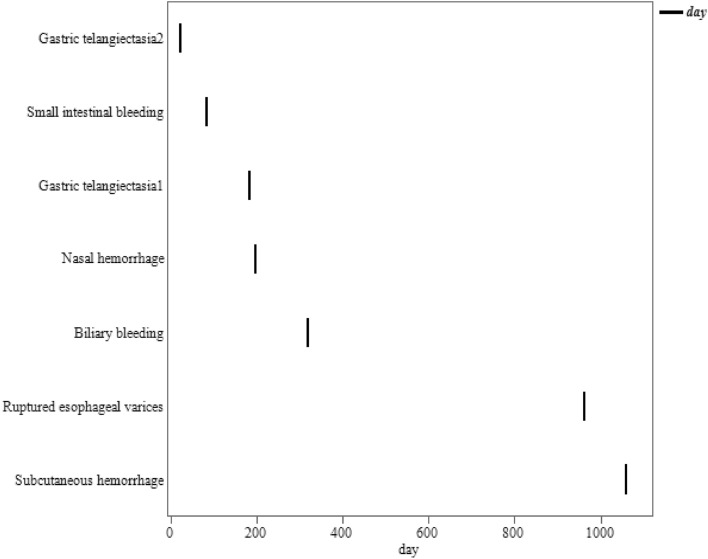
The timing of adverse events for edoxaban. The median time of occurrence of adverse events in the edoxaban group was 196 (132–641) days.

**Figure 7:**
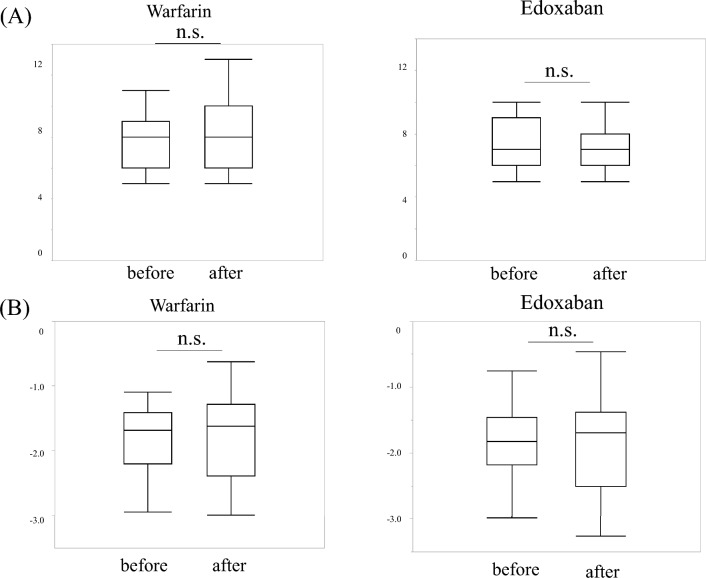
Changes in hepatic reserves before and after treatment of portal vein thrombosis. There was no change in the Child–Pugh score (**A**) or ALBI score (**B**) after 6 months of edoxaban or warfarin treatment. ALBI, albumin-bilirubin.

Adverse events in the warfarin group were observed in two patients (11.1%): one with epistaxis and one with liver damage. In contrast, seven patients (16.2%) in the edoxaban group had adverse events: four had gastrointestinal bleeding, one had biliary bleeding, one had subcutaneous bleeding, and one had epistaxis, all in the treatment-responsive group (Table 3). Based on the Bleeding Academic Research Consortium Definition for Bleeding classification^17^, there was one case of Type 2 bleeding in the warfarin group and four cases of Type 2 bleeding and three cases of Type 3a bleeding (transfusion performed) in the edoxaban group. The median time of occurrence of adverse events in the edoxaban group was 196 (132–641) days (Fig. 6). No significant differences were observed in the frequency of adverse events, and there were no deaths occurred due to adverse events. There was no change in the Child–Pugh or ALBI scores after 6 months of edoxaban or warfarin treatment (Fig. 7).

## Discussion

The present study demonstrated that treatment with edoxaban can achieve long-term recanalization as a single agent without compromising the hepatic reserves. We considered edoxaban, which is easy to administer and has a strong therapeutic effect, as a candidate primary agent for the treatment of PVT.

PVT is a major complication of cirrhosis and can be life threatening. However, there has been debate as to whether anticoagulation therapy should be administered, because approximately 40% of portal vein thrombi in patients with cirrhosis spontaneously dissolve^18^. Factors associated with spontaneous shrinkage of cirrhotic PVT include the absence of large collateral blood vessels (> 9 mm in diameter) and a low FIB-4 index^19^. However, the recurrence rate is high, estimated at 30%^20^. Recently, anticoagulation therapy was shown to reduce the risk of cirrhosis-related complications and improve survival in patients with cirrhotic PVT^21^. Therefore, PVT should eventually be treated with therapeutic interventions. A recent meta-analysis showed that anticoagulation does not significantly increase bleeding rates in patients with cirrhosis and PVT should be treated with anticoagulation therapy^22^.

Previously, anticoagulants, mainly heparin and warfarin, were used to treat PVT, and a certain degree of efficacy has been reported^1^. Heparin is an effective treatment that requires continuous injections, which are impractical for patients. Warfarin can be administered orally and can be discontinued quickly because antagonists are available. However, warfarin clearance is delayed in patients with cirrhosis because it depends on cytochrome P450-dependent pathways.

In addition, patients with a high PT-INR at baseline may be difficult to monitor^23^. It is also difficult to determine the optimal PT-INR value. AT III is the only drug approved for insurance in Japan for PVT, with an AT III activity below 70%, and its efficacy and safety are assured^24^. However, it is only a replacement therapy, and there is a high possibility of PVT recurrence due to a renewed decrease in AT III activity after treatment discontinuation. Continuous supplementation with AT III is not recommended because it is administered intravenously daily, requires hospitalization, and is expensive. In conclusion, AT III should only be used for initial treatment, and other maintenance therapies should be considered for relapse prevention.

Danaparoid sodium is a low-molecular-weight heparinoid (molecular weight: 5,500). An increasing number of reports have indicated that anticoagulation with danaparoid sodium is effective and safe for PVT in patients with cirrhosis^10^. It is characterized by a very high anti-Xa/thrombin activity ratio (22-fold; standard heparin is 1-fold) and a considerably long half-life in the blood (20 h; standard heparin is 0.5–1 h). Similar to standard and low-molecular-weight heparin, it exerts its anticoagulant activity in an AT III-dependent manner, primarily by blocking factor Xa. In Japan, it is only indicated for disseminated intravascular coagulation; however, in Europe, it is frequently used for heparin-induced thrombocytopenia and deep vein thrombosis, and good results have been reported. Danaparoid sodium must be administered intravenously, making it difficult to continue its use in outpatient settings. Furthermore, shipments of sodium danaparoid have recently been suspended, and there is no indication that the supply will resume. In addition, owing to its high recurrence rate, the question of how long PVT treatment should be continued is important. Currently, the American Association for the Study of Liver Diseases guidelines recommends anticoagulation for at least 3 months to allow recanalization and avoid worsening of intestinal infarction and portal hypertension^25^. Long-term maintenance treatment after the disappearance of PVT may be necessary to prevent recurrence. At the same time, close attention should be paid to serious complications, such as gastrointestinal bleeding.

Edoxaban is a direct-type Xa inhibitor with predictable pharmacokinetics^13,14^. Edoxaban is rapidly absorbed, reaching a peak blood concentration in 1–2 h. Approximately 50% of the absorbed dose is excreted by the kidneys^15^ and is not subject to hepatic metabolism^16^. Two recent studies^26,27^ concluded that edoxaban (administered once daily after initial treatment with heparin) could be an alternative to warfarin in patients with venous thromboembolism. In these studies, the therapeutic effect of edoxaban was comparable with that of standard high-quality therapies. Edoxaban has also been reported to cause significantly less bleeding in a wide range of patients with venous thromboembolism, including those with severe pulmonary embolism, suggesting that it may also be useful in patients with Child–Pugh B and C cirrhosis. The increased pharmacodynamic efficacy of another DOAC, rivaroxaban, in cirrhotic patients with moderate liver damage classified as Child–Pugh B and C was another reason for selecting edoxaban^28^. Edoxaban has been reported to be effective as maintenance therapy after danaparoid sodium administration^29^. We conducted this study to determine whether edoxaban is useful as a single agent when danaparoid sodium is not readily available and concluded that edoxaban is useful as a single agent in the treatment of PVT. The edoxaban dose is set at 60 mg daily if the patient weighs more than 60 kg and at 30 mg daily if the patient weighs less than 60 kg, has impaired renal function, or is taking concomitant medications that require caution. However, because the frequency of gastrointestinal bleeding was significantly lower with a 30 mg edoxaban regimen than with warfarin, we uniformly started with 30 mg in this study^30^. In addition, there have been some case reports of severe bleeding with 60 mg edoxaban use^31^. In this study, the 30 mg edoxaban regimen was sufficiently effective for PVT, with no fatal adverse events. Edoxaban is a potent inhibitor of factor Xa without mediating AT III and can achieve a stable therapeutic effect in patients with chronic liver disease with decreased AT III levels^16^. In addition, since there is no need to monitor PT-INR, and the hassle of intravenous infusion is avoided, it is an ideal treatment for long-term maintenance, with a lower patient burden. An additional advantage of edoxaban is that it can be administered as an outpatient drug. Sarcopenia is a problem in patients with chronic liver diseases such as cirrhosis and hepatocellular carcinoma^32,33^, and easy hospitalization may contribute to sarcopenia. Based on this study, we suggest starting edoxaban 30 mg monotherapy for PVT therapy, including acute onset, outpatients, patients with chronic PVT, patients with concomitant portal vein tumor plugs, and patients at a high risk of bleeding. However, in this study, there were cases of adverse events such as bleeding, so monitoring for coagulation abnormalities and periodic upper gastrointestinal endoscopy are necessary. The timing of adverse events is variable and requires constant vigilance during treatment.

This study had certain limitations. First, this was a retrospective study with a limited number of participants and period of observation. In addition, because the number of cases was small, there was variability in background factors. The warfarin group, in particular, had patients who could not achieve the therapeutic range due to coagulation abnormalities caused by cirrhosis. Therefore, after edoxaban was launched in Japan, more cases were treated with edoxaban. A large-scale prospective study is needed to determine whether the therapeutic effect of edoxaban is truly significant and safety. However, owing to its simplicity, edoxaban monotherapy may be an effective treatment option for PVT.

In conclusion, edoxaban monotherapy was effective for the treatment of PVT in patients with cirrhosis. Edoxaban monotherapy may improve the prognosis of patients with cirrhosis by achieving thromboprophylaxis without compromising reserves, is easily initiated in an outpatient setting, and may be an ideal first choice treatment for PVT.

## Methods

### Patients

The subjects were 76 patients with portal vein thrombosis and a background of liver cirrhosis who underwent therapeutic intervention between April 2012 and May 2021 at the Department of Gastroenterology, Kagawa University. Sixty-one patients treated with warfarin or edoxaban alone were included in the analysis, and those treated with danaparoid sodium or AT III were excluded. Data were collected on sex, age at thrombus onset, date of diagnosis, portal vein thrombus site (presence or absence of main portal vein occlusion), liver reserves, cirrhosis complications (ascites, hepatic encephalopathy, and esophageal varices), treatment method (drug type and duration), date of treatment initiation, treatment outcome, adverse events of treatment, changes in liver reserves after treatment, outcome, and cause of death. Cirrhosis was diagnosed comprehensively using criteria such as changes in liver morphology based on computed tomography (CT) imaging or abdominal ultrasonography, decreased platelet count (less than 140 × 10^9^/L), and severe fibrosis proven by liver biopsy. Patients with hepatitis B were defined as those who were positive for hepatitis B surface antigen or had a history of hepatitis B treatment. Patients with hepatitis C were defined as those who were positive for HCV antibody or HCV RNA or had a history of treatment for hepatitis C. Patients with non-B and non-C hepatitis were defined as those who did not fit into any of the above categories. Endoscopy was performed before treatment, and patients with varices who required treatment underwent endoscopic infusion sclerotherapy or endoscopic variceal ligation. The choice of treatment for PVT was determined by the physician in charge. Edoxaban (Lixiana; Daiichi-Sankyo, Tokyo, Japan) was initiated at a uniform dose of 30 mg because of the risk of bleeding in patients with cirrhosis. Warfarin was administered with a target prothrombin time-to-international normalized ratio (PT-INR) of 2.0–3.0.

### Definition of treatment responses

PVT was diagnosed by at least two radiologists, including a skilled radiologist with specialist qualifications. PVT was distinguished from portal vein tumor plug by dynamic CT with contrast agents. All patients underwent dynamic CT with contrast agents to evaluate the therapeutic effect of PVT. The diameter of the PVT was measured in the axial view of contrast-enhanced CT, which was evaluated as a percentage of the portal vein diameter by two physicians (a gastroenterologist and a radiologist). To establish the site of PVT, the portal system was evaluated separately for the main portal vein, left or right intrahepatic portal vein, superior mesenteric artery, and splenic vein. Treatment response was categorized as complete response (CR), partial response (PR), stable disease (SD), or progressive disease (PD). CR was defined as complete disappearance of PVT, PR was defined as ≥ 50% to < 100% reduction, SD was defined as < 50% reduction, and PD was defined as an increase in PVT volume after treatment compared to pre-treatment thrombus volume.

### Follow-up

All patients underwent clinically appropriate follow-up, including blood work within 1 month of the initiation of PVT treatment and thrombus assessment by dynamic CT within 6 months. Hepatic reserves were assessed 6 months after the start of PVT treatment using imaging studies. Patients who were followed up were evaluated for changes in PVT over time, prognosis, and adverse events of PVT treatment. In cases of adverse bleeding, edoxaban administration was paused for a short period until hemostasis was confirmed, and then it was resumed.

### Evaluation of liver functional reserve

The Child–Pugh classification and albumin-bilirubin (ALBI) grading system^34^ were used to assess changes in liver function reserves. The ALBI score was calculated based on serum albumin and total bilirubin values using the following formula: ALBI score = [log10 bilirubin (μmol/L) × 0.66] + [albumin(g/L) ×  − 0.085]. The ALBI grade was defined based on the following scores: ≤ − 2.60, grade 1; between − 2.60 and − 1.39, grade 2; and > − 1.39, grade 3. ALBI grade 2 was subdivided into 2a and 2b (referred to as modified ALBI grade) according to an ALBI score of − 2.27, which corresponds to a cutoff value of 15-min indocyanine green retention rate of 30%^35^.

### Statistical analysis

Continuous variables are presented as median values, with ranges shown in parentheses. Categorical variables are presented as numbers and percentages. Comparisons between the two groups were performed using the Wilcoxon rank sum test, chi-square test, and Fisher's exact probability test. Representative values are presented as medians (interquartile range). Survival analysis was performed using the Kaplan–Meier method or Cox proportional hazards model. Statistical significance was set at *p* < 0.05. All statistical analyses were performed using JMP Pro 17.0.0 (SAS Institute, Cary, NC, USA).

### Ethics statement

This study was conducted in compliance with the Declaration of Helsinki and the Ethical Guidelines for Clinical Trials in Medical Research Involving Human Subjects and was approved by the Ethics Committee of Kagawa University School of Medicine (Ethics Committee Reception Number 2019-256). The written informed consent was signed from all participants included in this study. The purpose and methods of the study were disclosed, and the participants were given the opportunity to refuse the use of their medical information at their request.

## Data Availability

All data generated or analysed during this study are included in this published article.

## Abbreviations

ALBI: Albumin-bilirubin
AT: Antithrombin
CR: Complete response
CT: Computed tomography
DOACs: Direct oral anticoagulants
HCC: Hepatocellular carcinoma
MST: Median survival time
ORR: Overall response rate
PD: Progressive disease
PR: Partial response
PT-INR: Prothrombin time-to-international normalized ratio
PVT: Portal vein thrombosis
SD: Stable disease
